# Methylphenidate Versus Placebo for Treating Fatigue in Patients With Advanced Cancer: Randomized, Double-Blind, Multicenter, Placebo-Controlled Trial

**DOI:** 10.1200/JCO.23.02639

**Published:** 2024-05-17

**Authors:** Patrick Charles Stone, Ollie Minton, Alison Richardson, Peter Buckle, Zinat E. Enayat, Louise Marston, Nick Freemantle

**Affiliations:** ^1^ Marie Curie Palliative Care Research Department, Division of Psychiatry, University College London (UCL), London, United Kingdom; ^2^ University Hospitals Sussex NHS Foundation Trust, Worthing Hospital, Lyndhurst Road, Worthing, West Sussex, United Kingdom; ^3^ University of Southampton & University Hospital Southampton NHS Foundation Trust, Southampton General Hospital, Southampton, United Kingdom; ^4^ Department of Primary Care & Population Health, Institute of Epidemiology & Health Care, Faculty of Population Health Sciences, University College London (UCL), London, United Kingdom; ^5^ Comprehensive Clinical Trials Unit, University College London (UCL), London, United Kingdom

## Abstract

**PURPOSE:**

To compare effects and side effects of 6 weeks of individually dose-titrated methylphenidate or placebo on fatigue in palliative care patients with advanced cancer.

**METHODS:**

This is a randomized, double-blind, placebo-controlled, multicenter trial. Eligible patients had advanced incurable cancer and fatigue >3/10. Principal exclusions were hypertension; psychiatric, cardiovascular, cerebrovascular, renal, liver, or blood disorders; substance dependency; and epilepsy. Patients were randomly assigned 1:1 methylphenidate or placebo starting at 5 mg twice daily. Dose of methylphenidate/placebo was titrated once per week, over 6 weeks, up to a maximum of 20 mg three times daily. Trial ended at 10 weeks. Primary outcome was the difference in Functional Assessment of Chronic Illness Therapy Fatigue (FACIT-F) scores between groups at 6 ± 2 weeks. Secondary outcomes included adverse effects, quality of life, and mood.

**RESULTS:**

One hundred sixty-two patients (73 men; mean, 65.8; standard deviation [SD], 10.3 years) were randomly assigned, and three were excluded from analysis. Seventy-seven were allocated placebo (baseline FACIT-F = 22 [SD, 10]); 82 were allocated methylphenidate (FACIT-F = 20 [SD, 9]). After 6 ± 2 weeks, FACIT-F scores were 1.97 points (95% CI, –0.95 to 4.90; *P* = .186) higher (better) on methylphenidate than placebo. Across 10 weeks of the study, FACIT-F was nominally higher in the methylphenidate group versus placebo (Diff, 2.20 [95% CI, 0.39 to 4.01]), but this did not reach the minimally clinically important difference (5-points). At 6 weeks, there were no differences between groups in quality-of-life or symptom domains except for depression scores (nominally reduced in the methylphenidate group: Diff, –1.35 [95% CI, –2.41 to –0.30]). There were no differences in mortality or serious adverse events.

**CONCLUSION:**

After 6 ± 2 weeks of treatment, methylphenidate was not superior to placebo for treating fatigue in advanced cancer. Methylphenidate was safe and well-tolerated.

## Teaser

Methylphenidate v placebo in advanced cancer: no clinically important improvement in fatigue

## INTRODUCTION

Cancer-related fatigue (CRF) is common and distressing.^1^ It is more prevalent and severe in patients with advanced cancer^2^ and those receiving palliative care.^3,4^ Treatment options are limited. Exercise has the best evidence of effectiveness;^5^ however, in patients with advanced cancer, this may not be feasible or realistic. Psychosocial interventions may help,^6^ but there is less evidence for effectiveness in advanced cancer.^7^

**CONTEXT**

**Key Objective**
To evaluate the clinical effectiveness and adverse effects of individually titrated doses of methylphenidate versus placebo for the relief of fatigue in patients with advanced cancer.
**Knowledge Generated**
Methylphenidate (in doses up to 20 mg three times/day) was no more effective than placebo at relieving fatigue after 6 (±2 weeks). Although methylphenidate had some measurable effects on fatigue at other timepoints, these improvements did not reach a level to be considered clinically important. Methylphenidate was safe to use and was well-tolerated in this group of patients.
**Relevance *(C. Zimmermann)***
While methylphenidate is generally well tolerated by patients with cancer receiving palliative care, its use is not supported by the available evidence. Further trials of methylphenidate should target different populations (eg, patients at earlier stages of disease) or other indications (eg, depression).*
*Relevance section written by *JCO* Associate Editor Camilla Zimmermann, MD, PhD, FRCPC.

Methylphenidate inhibits catecholamine reuptake, increases central dopamine and noradrenaline,^8^ and is sometimes used as a treatment for CRF. The National Comprehensive Cancer Network CRF guideline^9^ advises that methylphenidate can be considered in selected patients, but should be used cautiously and not until treatment and disease-specific morbidities have been characterized and excluded. Moreover, it notes that optimum dosing and schedule of methylphenidate remain uncertain. The European Society of Medical Oncology guideline on CRF is noncommittal about the issue.^10^

Belloni et al^11^ undertook a meta-review of pharmacologic interventions to improve CRF, including three systematic reviews^12-14^ and four meta-analyses of methylphenidate versus placebo. They reported a combined effect size for methylphenidate of –0.48 (95% CI, –0.75 to –0.21; *P* = .0004). Of nine clinical studies reported in the review, three reported benefit^15-17^ and six reported no benefit.^18-23^ More recently, Centeno et al^24^ reported no benefit after 6 days of methylphenidate (at doses of 10 mg at breakfast and 5 mg at other times, up to a maximum of 25 mg/day), whereas Pedersen et al^25^ reported that 10 mg of as-required methylphenidate was significantly better than placebo. Thus, although meta-analyses have reported moderate effect sizes in favor of methylphenidate, most individual studies have shown no benefit. The largest study to do so^15^ was undertaken in a population of patients with good performance status (93% Eastern Cooperative Oncology Group 1 or 2) and a mean of >2 years after completion of chemotherapy and who therefore probably did not have advanced disease (stage not reported). The study used relatively higher doses (equivalent to >50 mg/day [given in divided doses, either two or three times daily, at the discretion of the investigator] methylphenidate) administered over relatively longer periods (8 weeks), with greatest improvements occurring after 4 weeks of therapy. Only three other trials^19,20,22^ have used doses of methylphenidate ≥40 mg per day given once daily or in two divided doses (or equivalent), for at least 4 weeks.

In light of ongoing uncertainty about effectiveness of methylphenidate, this study was commissioned by the UK National Institute for Health Research Health Technology Assessment Programme. The primary aim was to compare fatigue scores in patients with advanced cancer receiving individually titrated doses of methylphenidate (up to 20 mg three times daily) versus placebo, after 6 weeks of treatment. Secondary aims were to compare other measures of quality of life, adverse events (AEs), activities of daily living, appetite, satisfaction of patients, survival, and the need for other medication.

## METHODS

### Trial Design

This was a double-blind, 1:1 randomized, placebo-controlled, parallel-group, multicenter trial. The trial was registered (ISRCTN 79478762 and EudraCT 2017-001950-33). The protocol was approved by the London City and East Research Ethics Committee (Ref 17/LO/0871), the Health Research Authority, and the Medicine and Healthcare products Regulatory Agency.

### Trial Modifications After Commencement

The trial started in June 2018. In October 2018, in response to below expected recruitment, inclusion and exclusion criteria were modified (these and subsequent changes are summarized in Protocol v14.0 January 17, 2023, available as supplementary information). Principal changes included reduction in minimum permissible estimated glomerular filtration rate (eGFR) from 60 mL/min to 45 mL/min and exclusion of patients with uncontrolled (rather than pre-existing) heart failure, angina, or drug dependency and with myocardial infarction or stroke within the previous year (rather than at any previous time). Recruitment to the trial was suspended from March to October 2020 because of the COVID-19 pandemic. Further modifications were then made, including greater use of remote (rather than face-to-face) follow-up assessments and merger of separate screening and enrollment visits.

### Participants

Eligible participants were adults with advanced incurable cancer (all tumor types), with fatigue >3/10 on a numerical rating scale (NRS), receiving generalist or specialist palliative care, with informed consent.

Exclusion criteria were as follows: pregnancy; breastfeeding; sensitivity to methylphenidate; glaucoma; pheochromocytoma; planned general anesthesia; concomitant psychostimulants, clonidine, warfarin, monoamine oxidase inhibitors, or modafinil; severe mood disorders; psychosis; hypertension (>160/100 mmHg); uncontrolled heart failure or angina; arterial occlusive disease; congenital heart disease; cardiomyopathies; myocardial infarction or stroke (within last year); life-threatening arrhythmias and channelopathies; cerebral aneurysm; cerebrovascular abnormalities; seizures; hyperthyroidism; hemoglobin <80 g/L; platelets <50 × 10^3^/μL; WBC count <1.5 × 10^9^/L; eGFR <45 mL/minute/1.73 m^2^; ALT > 2 or bilirubin > 1.5 × upper limit of normal; infection; substance/alcohol dependency; participation in another study; insufficient English language; and inability to swallow medication.

Participants were from 17 English palliative care services: 11 hospital-based, five hospices, and one community team.

### Interventions

Participants received methylphenidate 5 mg tablets or identical placebo tablets (Mawdsley-Brooks & Co Ltd, Doncaster, United Kingdom). There is no consensus about dose of methylphenidate for CRF.^9^ Participants started on one tablet twice daily (10 mg/day). Doses were individually titrated by principal investigators (according to participants' perceived efficacy and/or adverse effects), weekly for 6 weeks, up to maximum of four tablets, three times/daily (60 mg/day). Participants remained at week 6 dose for 2 weeks (maintenance phase). For all participants, doses were tapered after week 8 assessment and stopped completely for 1 week before the end of trial at week 10.

### Outcomes

Primary outcome was fatigue at 6 (±2) weeks measured by 13-item Functional Assessment of Chronic Illness Fatigue (FACIT-F) questionnaire.^26^ Scores range between 0 and 52, with higher scores representing lower levels of fatigue. For primary outcome assessment, in cases where FACIT-F data were missing at week 6, assessments at 2 weeks either side were permitted.

Secondary outcomes were as follows: other measures of quality of life, AEs, activities of daily living, appetite, anxiety, depression, satisfaction of patients, survival, need for other medication, and fatigue at other times. Secondary outcomes were assessed at 3, 6, and 10 weeks (± 4 days) except for fatigue and patient satisfaction, which were measured weekly; need for further medication assessed at week 6; and survival over trial period.

Secondary outcomes were assessed using (1) European Organization for Research and Treatment of Cancer Palliative care Quality of Life Questionnaire (EORTC QLQ-C15-PAL)^27,28^ comprising 15 items covering quality of life, physical and emotional functioning, pain, fatigue, nausea, anorexia, dyspnea, constipation, and insomnia. Higher scores represent greater severity on symptom scales and better functioning on functional/quality-of-life scales; (2) EQ-5D-5L^29^ comprising questions on mobility, self-care, usual activities, pain/discomfort, and anxiety/depression; (3) five-point Global Benefit Score of patient satisfaction with fatigue treatment; (4) 14-item Hospital Anxiety and Depression Scale (HADS)^30^ consisting of anxiety (HADS-A) and depression (HADS-D) subscales with higher scores representing worse symptoms; (5) AEs elicited weekly from patients (and graded according to severity as mild, moderate, or severe) in response to the list of known potential AEs. Serious adverse events (SAEs) were reported and recorded separately.

### Sample Size

With 162-230 randomly assigned and 130-172 evaluable patients (20%-25% attrition), this study had 80%-90% power to detect five-point difference on FACIT-F (effect size 0.5) at 6 ± 2 weeks between groups at 5% significance (two sided). On the basis of previous research,^31-33^ this was regarded as a minimal clinically important difference (MCID).

### Random Assignment and Concealment

Random assignment (1:1) was performed using an online platform^34^ with random permuted blocks, stratified by site, receipt of disease-modifying therapy, high depression score (>10 on HADS-D), and fatigue severity (>7/10 on the NRS). Data were analyzed blind to allocation.

### Statistical Methods

Analysis was by intention to treat. Participants ingested at least one correctly allocated tablet. Data were analyzed using Stata^35^ and SAS.^36^ Summary statistics for continuous variables were reported as mean and standard deviation (SD) or median and IQR, according to distribution. Categorical variables were reported as frequency (%).

Analysis of primary outcome used a mixed effects linear model. Each participant provided two values, one for baseline and the other for follow-up, with random intercepts for participant. Fixed effects were time point (baseline or follow-up), randomized treatment, and stratification factors. For analyses with FACIT-F as outcome, the fatigue stratification factor was not included because baseline fatigue was already included in the models. For all analyses, the site stratification factor was not included as this did not result in good model fit possibly because some sites had few participants. The denominator *df* for test for treatment effect was derived from the number of participants. We report *P* values for the randomized group with estimate and 95% CI. The primary outcome was also assessed across three subgroup analyses (the stratification variables). For analyses of interaction between stratification factors and randomized groups, all stratification factors (with exception of site) were included.

Secondary outcomes were analyzed in a similar way to the primary outcome using mixed effects linear regression. Where HADS-D was the outcome, the depression stratification variable was not included because baseline depression was already included in the model. Survival of participants was analyzed using Cox proportional hazards regression. Global Benefit Score dichotomized as stayed the same or got worse versus got better was analyzed using logistic regression, including the randomized group only, because of the lack of power. Frequency and percentage of SAEs and severe and other AEs were analyzed descriptively. No *P* values were calculated for secondary outcome statistical modeling; estimates and 95% CI are reported.

Most participants had multiple observations of FACIT-F, so we undertook an analysis incorporating all available data longitudinally using mixed effects linear regression. We examined the sensitivity of the primary outcome to allocation of participants who were randomly assigned in error, repeating the primary analysis including them in their allocated group. In addition, we undertook a threshold analysis, in which participants with missing data in the methylphenidate group were attributed a score at highest 10% of placebo values and participants with missing data in the placebo group were attributed median value for placebo participants.

## RESULTS

**FIG 1.**
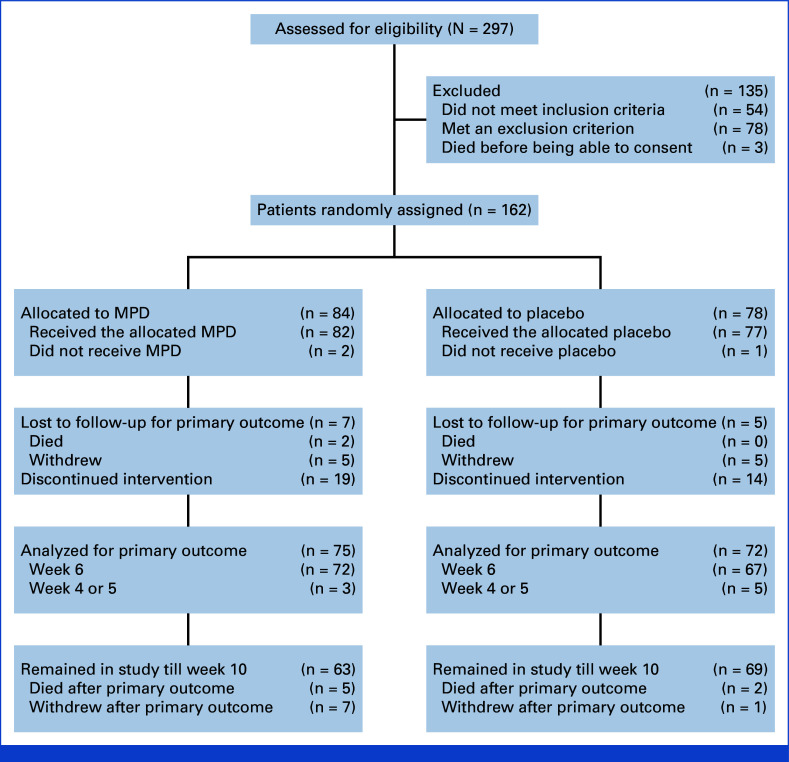
CONSORT diagram showing the flow of study participants. MPD, methylphenidate.

Between June 29, 2018, and April 27, 2023, 162 participants were randomly assigned and 159 were included in analyses (Fig 1); 73 men and 86 women; mean age 63.7 years (SD, 11.9). The last visit of the last patient was July 3, 2023. Screening and baseline data for participants included in analyses are shown in Tables 1 and 2. Participant groups were balanced with regard to baseline characteristics. Table 3 shows comparison of characteristics in each group after 6 weeks of dose titration. At week 6, the median daily dose attained in each group was 6 tablets per day (=methylphenidate 15 mg twice daily) tablets. A similar proportion of participants in each group considered trial medication/placebo to be effective (methylphenidate 27 of 72, 37.5%; placebo 23 of 67, 34%). Fatigue, anxiety, depression, quality-of-life, and other symptom scores and blood pressure and pulse rates were similar between groups.

**TABLE 1.** Screening Data of Analyzed Participants

Characteristic	Placebo	Methylphenidate
Male, n/N (%)	35/77 (45)	38/82 (46)
Age at random assignment, years, mean (%)	62.6 (11.8)	64.7 (11.9)
ECOG performance status, No. (%)		
0	3 (4)	3 (4)
1	30 (39)	38 (46)
2	27 (35)	21 (26)
3	3 (4)	5 (6)
4	0 (0)	0 (0)
Missing data	14 (18)	15 (18)
Primary diagnosis, No. (%)		
Breast	20 (26)	21 (26)
Lung	15 (20)	11 (14)
Prostate	14 (18)	9 (11)
Lower GI	7 (9)	11 (14)
Urogenital	5 (7)	5 (6)
Upper GI	4 (5)	5 (6)
Other	3 (4)	3 (4)
Hematologic	2 (3)	6 (7)
Gynecologic	2 (3)	5 (6)
Head and neck	0 (0)	1 (1)
Neurologic	1 (1)	1 (1)
Unknown primary	1 (1)	0 (0)
Two primary diagnoses	1 (1)	3 (4)
Rare tumor groups	0 (0)	0 (0)
Missing data	2 (3)	1 (1)
Sites of metastases, No. (%)		
Bone	32 (42)	31 (38)
Lung	23 (30)	20 (25)
Nodal	17 (22)	17 (21)
Liver	12 (16)	16 (20)
Other	11 (14)	15 (19)
None	6 (8)	6 (7)
Adrenal	2 (3)	3 (4)
Malignant pleural effusion	2 (3)	1 (1)
Renal	1 (1)	2 (2)
Brain	1 (1)	2 (2)
Malignant ascites	0 (0)	1 (1)
Unknown	0 (0)	1 (1)
Missing data	1 (1)	1 (1)
Blood results		
Thyroxine, pmol/L, mean (SD)	14.7 (2.9)	15.1 (3.2)
Thyroid-stimulating hormone, mU/L, median (IQR)	1.7 (1.1 to 2.5)	1.8 (1.3 to 3.0)
Hemoglobin, g/L, mean (SD)	121 (17)	123 (15)
Platelets, ×10^9^/L, median (IQR)	237 (189 to 310)	248 (206 to 298)
WBC, ×10^9^/L, median (IQR)	6.2 (4.6 to 7.9)	5.8 (4.4 to 7.3)
eGFR, mL/min/1.73 m^2^, median (IQR)	72 (60 to 90)	69 (60 to 90)
ALT, U/L, median (IQR)	16 (12 to 31)	18 (13 to 29)
Bilirubin, µmol/L, median (IQR)	7 (5 to 9)	6 (5 to 10)
Stratification factors, No. (%)		
Current, recent, or scheduled disease-modifying treatment	62 (81)	62 (76)
Depression >10 on HADS-D	17 (22)	25 (30)
Fatigue >7/10 on NRS	26 (34)	30 (37)

Abbreviations: ECOG, Eastern Cooperative Oncology Group; EGFR, estimated glomerular filtration rate; HADS-D, Hospital Anxiety and Depression Scale-Depression; NRS, numerical rating scale; SD, standard deviation.

**TABLE 2.** Baseline Data by Randomized Group

Characteristic	Placebo	Methylphenidate
FACIT-F, mean (SD)	22 (10)	20 (9)
HADS depression score, median (IQR)	6 (4-9)	7 (5-11)
HADS anxiety score, median (IQR)	4 (2-9)	6 (3-9)
EORTC QLQ-C15-PAL, mean (SD)		
Pain	53 (20)	52 (19)
Physical functioning	50 (17)	50 (18)
Emotional functioning	42 (17)	44 (17)
Fatigue	72 (18)	74 (15)
Quality of life	31 (14)	30 (13)
Nausea	39 (22)	44 (22)
Loss of appetite	52 (26)	52 (25)
Shortness of breath	53 (24)	52 (21)
Constipation	41 (21)	47 (24)
Sleep	51 (27)	56 (25)
EQ-5D-5L utility, mean (SD)	0.65 (0.18)	0.62 (0.21)
Systolic blood pressure, mean (SD)	126 (16)	130 (16)
Diastolic blood pressure, mean (SD)	76 (10)	77 (10)
Pulse rate, mean (SD)	81 (13)	83 (13)
Concomitant medication, No. (%)		
Strong opioids	31 (40)	31 (38)
Other analgesia	39 (51)	34 (41)
Benzodiazepines	5 (6)	4 (5)
Antidepressants	22 (29)	32 (39)
Steroids	16 (21)	21 (26)
None of the above	4 (5)	2 (2)

Abbreviations: EORTC QLQ-C15-PAL, European Organization for Research and Treatment of Cancer Quality of Life Questionnaire Core 15 Palliative Care; FACIT-F, Functional Assessment of Chronic Illness Therapy—Fatigue; HADS, Hospital Anxiety and Depression Scale; SD, standard deviation.

**TABLE 3.** Week 6 Data by Randomized Group

Characteristic	Placebo	Methylphenidate
Dose level achieved, No. (%)		
0 tablets	13 (19)	11 (15)
2 tablets	7 (10)	6 (8)
4 tablets	7 (10)	11 (15)
6 tablets	10 (15)	13 (18)
8 tablets	6 (9)	15 (21)
10 tablets	3 (4)	3 (4)
12 tablets	21 (31)	14 (19)
Participant feels…, No. (%)		
Trial medication is effective	23/67 (34)	27/72 (38)
Overall same or worse	38/67 (57)	45/72 (63)
FACIT-F, 6 weeks only, mean (SD)	(n = 67) 31 (12)	(n = 72) 33 (11)
FACIT-F, 6 ± 2 weeks, mean (SD)	(n = 72) 31 (12)	(n = 75) 32 (11)
HADS scores	n = 66	n = 72
Depression	5 (3 to 9)	4 (2 to 8)
Anxiety	4 (2 to 6)	4 (2 to 6)
EORTC QLQ-C15-PAL, mean (SD)	n = 66	n = 72
Pain	45 (18)	46 (18)
Physical functioning	44 (17)	41 (16)
Emotional functioning	35 (15)	38 (17)
Fatigue	55 (19)	54 (17)
Quality of life	28 (14)	26 (11)
Nausea	35 (19)	41 (19)
Loss of appetite	41 (23)	47 (26)
Shortness of breath	51 (26)	45 (23)
Constipation	36 (19)	43 (22)
Sleep	47 (25)	44 (24)
EQ-5D-5L utility, mean (SD)	(n = 66) 0.70 (0.22)	(n = 72) 0.71 (0.23)
Blood pressure	n = 63	n = 67
Systolic blood pressure, mean (SD)	127 (16)	128 (20)
Diastolic blood pressure, mean (SD)	77 (9)	78 (12)
BP >160/100 mmHg, No. (%)	2 (3)	2 (3)
Pulse rate bpm, mean (SD)	(n = 60) 85 (14)	(n = 67) 82 (12)
Increased or started…, n/N (%)		
Strong opioids	12/76 (16)	7/82 (9)
Other analgesia	4/77 (5)	7/82 (9)
Benzodiazepines	2/76 (3)	1/81 (1)
Antidepressants	2/77 (3)	4/81 (5)
Steroids	9/76 (12)	5/82 (6)

Abbreviations: EORTC QLQ-C15-PAL, European Organization for Research and Treatment of Cancer Quality of Life Questionnaire Core 15 Palliative Care; FACIT-F, Functional Assessment of Chronic Illness Therapy—Fatigue; HADS, Hospital Anxiety and Depression Scale; SD, standard deviation.

### Analysis of Primary Outcome

There was a nonsignificant reduction in fatigue (increased FACIT-F) in the intervention group (FACIT-F coefficient, 1.97 [95% CI, –0.95 to 4.90]; n = 159; *P* = .186) at 6 ± 2 weeks (Table 4). Sensitivity analysis similarly showed a nonsignificant difference between groups (FACIT-F coefficient, 2.05 [95% CI, –0.85 to 4.95]; n = 161). Threshold analysis was nominally significant although point estimate was still less than MCID (FACIT-F coefficient, 3.15 [95% CI, 0.26 to 6.04]; n = 159).

**TABLE 4.** Results of Statistical Analyses for Primary and Secondary Outcomes

Outcome	Estimate	95% CI
Primary outcome		
FACIT-F (coefficient), week 6 (±2), n = 159	1.97	–0.95 to 4.90
Stratification factors		
Disease-modifying treatment, yes, n = 35	0.12	–6.20 to 6.43
Disease-modifying treatment, no, n = 124	2.40	–0.77 to 5.58
*P* value for interaction	.002	
Baseline HADS-D >10, yes, n = 117	2.31	–1.05 to 5.67
Baseline HADS-D >10, no, n = 42	0.76	–5.42 to 6.95
*P* value for interaction	.552	
Fatigue >7/10 on NRS, yes, n = 56	1.28	–4.12 to 6.67
Fatigue >7/10 on NRS, no, n = 103	2.32	–1.01 to 5.66
*P* value for interaction	.067	
Secondary outcomes		
FACIT-F (coefficient), n = 159		
Week 1	0.06	–2.41 to 2.53
Week 2	3.25	0.53 to 5.97
Week 3	3.24	0.37 to 6.11
Week 4	3.16	0.16 to 6.16
Week 5	3.18	0.21 to 6.15
Week 6	3.11	0.16 to 6.05
Week 7	2.72	–0.51 to 5.94
Week 8	3.47	0.40 to 6.54
Week 9	1.91	–1.12 to 4.95
Week 10	–0.55	–3.56 to 2.47
All weeks	2.20	0.39 to 4.01
EORTC QLQ-C15-PAL (coefficients)		
Pain, n = 159		
Week 3	0.19	–4.85 to 5.24
Week 6	1.63	–3.66 to 6.93
Week 10	1.77	–3.96 to 7.50
Physical functioning, n = 159		
Week 3	–0.31	–4.05 to 3.44
Week 6	–2.67	–7.28 to 1.94
Week 10	1.70	–2.93 to 6.34
Emotional functioning, n = 158		
Week 3	0.75	–3.97 to 5.48
Week 6	1.71	–3.18 to 6.59
Week 10	3.40	–2.04 to 8.84
Fatigue, n = 159		
Week 3	–4.74	–9.78 to 0.29
Week 6	–2.50	–7.79 to 2.80
Week 10	2.86	–2.69 to 8.41
Quality of life, n = 159		
Week 3	0.71	–2.87 to 4.30
Week 6	–2.09	–5.91 to 1.74
Week 10	–0.25	–4.20 to 3.69
Nausea, n = 159		
Week 3	4.05	–2.22 to 10.31
Week 6	4.30	–2.07 to 10.66
Week 10	6.14	–0.53 to 12.82
Appetite, n = 159		
Week 3	0.85	–7.72 to 6.03
Week 6	6.71	–0.59 to 14.00
Week 10	4.04	–3.61 to 11.68
Shortness of breath, n = 159		
Week 3	0.03	–6.06 to 6.11
Week 6	–6.17	–12.78 to 0.44
Week 10	–4.71	–11.59 to 2.17
Constipation, n = 158		
Week 3	3.24	–3.25 to 9.74
Week 6	4.64	–2.02 to 11.29
Week 10	0.41	–6.36 to 7.18
Sleep, n = 159		
Week 3	–6.55	–13.74 to 0.64
Week 6	–5.49	–12.96 to 1.98
Week 10	–6.85	–13.97 to 0.28
EQ-5D-5L utility		
Week 3 (n = 147)	0.025	–0.027 to 0.076
Week 6 (n = 138)	0.028	–0.031 to 0.086
Week 10 (n = 130)	0.001	–0.052 to 0.055
Overall difference in QALYs (n = 159)	0.009	–0.006 to 0.024
HADS anxiety (n = 159)		
Week 3	–0.70	–1.61 to 0.22
Week 6	–0.39	–1.40 to 0.62
Week 10	–0.32	–1.42 to 0.77
HADS depression (n = 159)		
Week 3	–0.73	–1.65 to 0.19
Week 6	–1.35	–2.41 to –0.30
Week 10	–0.39	–1.45 to 0.66
Global benefit score (OR)		
Week 1 (n = 157)	0.68	0.34 to 1.34
Week 2 (n = 152)	0.77	0.41 to 1.45
Week 3 (n = 147)	0.80	0.42 to 1.55
Week 4 (n = 147)	0.66	0.34 to 1.26
Week 5 (n = 140)	1.48	0.76 to 2.88
Week 6 (n = 139)	1.27	0.64 to 2.51
Week 7 (n = 135)	1.06	0.53 to 2.12
Week 8 (n = 134)	1.31	0.64 to 2.70
Week 9 (n = 129)	1.29	0.62 to 2.70
Week 10 (n = 132)	2.11	0.96 to 4.61
Died at any time (OR) (n = 159)	0.86	0.45 to 1.61
Time to death (HR) (n = 159)	0.98	0.66 to 1.47
Increased or started… (OR)		
Strong opioids (n = 158)	0.50	0.18 to 1.34
Other analgesia (n = 159)	1.70	0.48 to 6.07
Benzodiazepines (n = 157)	0.46	0.04 to 5.21
Antidepressants (n = 158)	1.95	0.35 to 10.95
Steroids (n = 158)	0.48	0.15 to 1.51

Abbreviations: EORTC QLQ-C15-PAL, European Organization for Research and Treatment of Cancer Quality of Life Questionnaire Core 15 Palliative Care; FACIT-F, Functional Assessment of Chronic Illness Therapy—Fatigue; HADS, Hospital Anxiety and Depression Scale; HR, hazard ratio; NRS, numerical rating scale; OR, odds ratio.

### Interaction Between Stratification Factors and the Randomized Group

No subgroups (high/low baseline fatigue, high/low baseline depression, or disease-modifying therapies yes/no) showed significant improvements in FACIT-F. There was, however, significant interaction between disease-modifying treatment and primary outcome.

### Analyses of Secondary Outcomes

There were nominally significant increases in FACIT-F (lower fatigue) at all study weeks, except for week 1, week 7 (first week maintenance), week 9 (dose tapering), and week 10 (end of trial). There was a nominally significant reduction in fatigue across the 10-week period (2.20 [95% CI, 0.39 to 4.01]). No differences between groups in quality-of-life or symptom domains, except for HADS-Depression at week 6 (nominally reduced in the methylphenidate group: Diff, –1.35 [95% CI, –2.41 to –0.30]; Table 4), were observed.

### Harms

Table 5 shows the number of participants experiencing self-rated severe AEs at any time over the 10-week study period, by randomized group. There was no pattern to suggest increased adverse effects in the intervention group. In particular, no increased frequency of severe anxiety, insomnia, loss of appetite, or feelings of heart racing might have been expected with methylphenidate. There were 25 SAEs in 20 participants receiving methylphenidate and 25 SAEs among 16 participants receiving placebo. No SAEs were considered to be probably related to methylphenidate. There were no Suspected Unexpected Serious Adverse Reactions. Two participants in the placebo group and six participants in the methylphenidate group died within 75 days of random assignment (Fisher's exact *P* value .278).

**TABLE 5.** Number of Participants Experiencing Self-Rated Severe Adverse Event Any Time Over the 10 Weeks by Randomized Group

AE	Placebo (n = 76), No. (%)	Methylphenidate (n = 82), No. (%)
Cough	8 (11)	5 (6)
Sore throat	7 (9)	1 (1)
Other airways symptoms	12 (16)	11 (13)
Abdominal pain	11 (14)	7 (9)
Diarrhea	5 (7)	10 (12)
Nausea	5 (7)	7 (9)
Vomiting	2 (3)	1 (1)
Dry mouth	13 (17)	10 (12)
Other stomach/bowel symptoms	12 (16)	8 (10)
Anxiety	8 (11)	5 (6)
Depression	5 (7)	1 (1)
Irritability	6 (8)	3 (4)
Aggression	2 (3)	1 (1)
Mood swings	4 (5)	1 (1)
Abnormal behavior	1 (1)	1 (1)
Other mood or mental state symptoms	2 (3)	1 (1)
Hair loss	2 (3)	2 (2)
Itch	2 (3)	2 (2)
Skin rashes	4 (5)	1 (1)
Other skin or hair symptoms	4 (5)	2 (2)
Loss of appetite	14 (18)	11 (13)
Lost weight	0 (0)	4 (5)
Heart racing	2 (3)	2 (2)
Abnormal heart rhythms	1 (1)	0 (0)
Headache	8 (11)	3 (4)
Felt dizzy	7 (9)	7 (9)
Felt drowsy	17 (22)	9 (11)
Difficulty in sleeping	16 (21)	14 (17)
Abnormal muscle movements	3 (4)	2 (2)
Been abnormally active	1 (1)	1 (1)
Joint pain	12 (16)	7 (9)
Fever	2 (3)	3 (4)
Cold or flu-like symptoms	4 (5)	3 (4)

NOTE. One participant in the placebo group withdrew from the study before week 1 assessment and so did not provide any data about adverse effects.

Abbreviation: AE, adverse event.

## DISCUSSION

Six weeks of methylphenidate (up to 20 mg three times daily) was not superior to placebo for fatigue in patients with advanced cancer but was safe and well tolerated. These results align with other studies that have found no significant benefit for methylphenidate over placebo in patients with advanced cancer.^18-21,23,24^ Our main finding that methylphenidate did not improve fatigue was further supported by the lack of effect on other symptoms or on overall quality of life.

Our study had several strengths including individualized dose titration to a maximum tolerated dose, greater than 2-month duration, advanced cancer population, good retention of participants, close (once weekly) monitoring of AEs, and use of MCID in fatigue as the primary outcome. Our study was designed to reflect the way in which methylphenidate might be used in real-world clinical practice. Study participants were started at low daily doses and evaluated weekly, with doses of medication being titrated according to perceived effectiveness and/or adverse effects. We achieved excellent retention of trial participants and median daily doses of 15 mg twice daily (with many reaching maximum permitted doses of 20 mg three times daily). Conducting multicenter research studies in palliative care populations presents significant practical and logistical difficulties,^37-40^ and our study was also severely hampered by the COVID-19 pandemic. Despite this, we recruited sufficient participants to ensure that the study was adequately powered to address the research question.

The study by Lower et al,^15^ using the D-isomer of methylphenidate rather than racemic drug,^41^ reported that a dose equivalent to 55.4 mg (in two or three divided doses per day) of methylphenidate was effective at relieving CRF. Thus, it may be argued that the dose of methylphenidate attained in our study (median 15 mg twice daily) was too low to be effective. However, our protocol allowed for dose escalation up to a maximum of 20 mg three times daily for participants who were able to tolerate such doses (and many participants did so). Another potential limitation of our study was that (despite strict eligibility criteria) there were many potentially confounding variables (heterogenous cancers, treatment modalities, rates of disease progression, and polypharmacy), which might have obscured any effects of the intervention. We did not record reasons why concomitant medications were started or stopped throughout the trial. In line with other studies,^42^ we found a relatively large placebo response and this might have made the task of demonstrating effects of the intervention more difficult. The HADS, although validated for use in patients with advanced cancer,^43^ might not have been the best tool to assess psychological symptoms in this population.^44^

Because of potential for adverse effects, we applied stringent inclusion and exclusion criteria, meaning that trial participants were highly selected. Many palliative care patients at participating centers were not considered for inclusion because they had clear contraindications or else were too ill to participate. Over 58 months and across 17 sites, only 297 participants were formally screened, of whom only half were eventually randomly assigned. This suggests that, even if methylphenidate had been found to be effective in the trial population, its usefulness in day-to-day clinical practice might be questionable. Nonetheless, we managed to recruit a sample of palliative care patients with advanced cancer with a variety of different primary tumors and sites of metastases—who were broadly representative of the types of patients who might be considered for pharmacotherapy for fatigue.

There is no consensus about the MCID of FACIT-F. Reddy et al^32^ calculated MCID by comparing FACIT-F and subjective Global Benefit Scores using pooled data from three clinical trials in palliative care patients with advanced cancer. They reported that an increase of ≥10 points should be considered to represent the MCID because patients experienced this as representing a change that was moderately important, consistently beneficial. Other authors^31,33^ have argued for lower MCIDs varying between 2 and 5 points (integer values). However, these estimates used anchor-based (hemoglobin levels, performance status, and treatment response) and/or distribution-based (SDs and SEs) methods which may not be as relevant as patient-rated subjective comparators for this population. Nonetheless, to be conservative, rather than using the 10-point MCID proposed by Reddy et al, we powered our study using an MCID of only five points.

Having met our random assignment targets, we are confident that there was no statistically significant difference in primary outcome (fatigue at 6 ± 2 weeks) according to the treatment arm. Moreover, confidence intervals around our primary outcome excluded even an MCID between arms.

By contrast, most secondary fatigue outcomes (FACIT-F scores at other timepoints and across the 10-week period) were nominally significantly in favor of the intervention arm. The biggest change (FACIT-F coefficient, 3.47 [95% CI, 0.40 to 6.54]) was found at week 8 assessment. It is noteworthy that, even in this case, the point estimate for difference in fatigue scores was less than the MCID the trial had been established to detect. One secondary outcome (HADS-Depression) also showed a nominally significant change at week 6. All nominally significant differences in secondary outcomes should be regarded as exploratory findings as the study was not powered to look for such differences and no correction was made for multiple analyses.

We had anticipated that, even if methylphenidate was found to be effective, its usefulness might have been limited by risk of adverse effects. However, despite active elicitation of potential side effects and monitoring of blood pressure, we did not detect patterns of increased symptomatology in the treatment arm. This adds to limited safety data related to the use of methylphenidate in older patients with advanced cancer.^14,45^

On the basis of our findings, we do not recommend the use (or further trials) of methylphenidate for fatigue in patients with advanced cancer receiving palliative care. However, given the absence of major AEs, it would be safe to continue to explore its use in future clinical trials for other symptoms (eg, low mood or opioid-related drowsiness), in combination with other interventions (eg, exercise or psychological therapies) or in different populations (eg, post-treatment fatigue).
